# Tensor modeling of MRSA bacteremia cytokine and transcriptional patterns reveals coordinated, outcome-associated immunological programs

**DOI:** 10.1093/pnasnexus/pgae185

**Published:** 2024-05-04

**Authors:** Jackson L Chin, Zhixin Cyrillus Tan, Liana C Chan, Felicia Ruffin, Rajesh Parmar, Richard Ahn, Scott D Taylor, Arnold S Bayer, Alexander Hoffmann, Vance G Fowler, Elaine F Reed, Michael R Yeaman, Aaron S Meyer, with the MRSA Systems Immunobiology Group, Parmar Rajesh, Richard Ahn, Arnold S Bayer, Liana Chan, Yu-Ling Chang, Scott G Filler, Vance G Fowler, David Gjertson, Alexander Hoffmann, Felix Medie, Simon Mitchell, Elaine F Reed, Maura Rossetti, Felicia Ruffin, Yan Qin, Batu Sharma, Katherine Sheu, Joshua Thaden, Alan J Waring, Yan Q Xiong, Ying Zheng, Michael R Yeaman

**Affiliations:** Department of Bioengineering, University of California, Los Angeles, Los Angeles, CA 90024, USA; Bioinformatics Interdepartmental Program, University of California, Los Angeles, Los Angeles, CA 90024, USA; The Lundquist Institute for Biomedical Innovation, Harbor-UCLA Medical Center, Torrance, CA 90502, USA; Department of Medicine, David Geffen School of Medicine, University of California, Los Angeles, CA 90095, USA; Division of Infectious Diseases, Department of Medicine, Harbor-UCLA Medical Center, Torrance, CA 90502, USA; Division of Molecular Medicine, Department of Medicine, Harbor-UCLA Medical Center, Torrance, CA 90502, USA; Division of Infectious Diseases, Duke University School of Medicine, Durham, NC 27710, USA; Department of Pathology and Laboratory Medicine, University of California, Los Angeles, Los Angeles, CA 90095, USA; Institute for Quantitative and Computational Biosciences, David Geffen School of Medicine at UCLA, Los Angeles, CA 90095, USA; Department of Bioengineering, University of California, Los Angeles, Los Angeles, CA 90024, USA; The Lundquist Institute for Biomedical Innovation, Harbor-UCLA Medical Center, Torrance, CA 90502, USA; Department of Medicine, David Geffen School of Medicine, University of California, Los Angeles, CA 90095, USA; Institute for Quantitative and Computational Biosciences, David Geffen School of Medicine at UCLA, Los Angeles, CA 90095, USA; Division of Infectious Diseases, Duke University School of Medicine, Durham, NC 27710, USA; Department of Pathology and Laboratory Medicine, University of California, Los Angeles, Los Angeles, CA 90095, USA; The Lundquist Institute for Biomedical Innovation, Harbor-UCLA Medical Center, Torrance, CA 90502, USA; Department of Medicine, David Geffen School of Medicine, University of California, Los Angeles, CA 90095, USA; Division of Infectious Diseases, Department of Medicine, Harbor-UCLA Medical Center, Torrance, CA 90502, USA; Division of Molecular Medicine, Department of Medicine, Harbor-UCLA Medical Center, Torrance, CA 90502, USA; Division of Infectious Diseases, Duke University School of Medicine, Durham, NC 27710, USA; Department of Bioengineering, University of California, Los Angeles, Los Angeles, CA 90024, USA; Bioinformatics Interdepartmental Program, University of California, Los Angeles, Los Angeles, CA 90024, USA; Jonsson Comprehensive Cancer Center, University of California, Los Angeles, Los Angeles, CA 90024, USA; Eli and Edythe Broad Center of Regenerative Medicine and Stem Cell Research, University of California, Los Angeles, Los Angeles, CA 90024, USA

**Keywords:** MRSA, persistence, tensor factorization, CD4+ T cells, granulocytes

## Abstract

Methicillin-resistant *Staphylococcus aureus* (MRSA) bacteremia is a common and life-threatening infection that imposes up to 30% mortality even when appropriate therapy is used. Despite in vitro efficacy determined by minimum inhibitory concentration breakpoints, antibiotics often fail to resolve these infections in vivo, resulting in persistent MRSA bacteremia. Recently, several genetic, epigenetic, and proteomic correlates of persistent outcomes have been identified. However, the extent to which single variables or their composite patterns operate as independent predictors of outcome or reflect shared underlying mechanisms of persistence is unknown. To explore this question, we employed a tensor-based integration of host transcriptional and cytokine datasets across a well-characterized cohort of patients with persistent or resolving MRSA bacteremia outcomes. This method yielded high correlative accuracy with outcomes and immunologic signatures united by transcriptomic and cytokine datasets. Results reveal that patients with persistent MRSA bacteremia (PB) exhibit signals of granulocyte dysfunction, suppressed antigen presentation, and deviated lymphocyte polarization. In contrast, patients with resolving bacteremia (RB) heterogeneously exhibit correlates of robust antigen-presenting cell trafficking and enhanced neutrophil maturation corresponding to appropriate T lymphocyte polarization and B lymphocyte response. These results suggest that transcriptional and cytokine correlates of PB vs. RB outcomes are complex and may not be disclosed by conventional modeling. In this respect, a tensor-based integration approach may help to reveal consensus molecular and cellular mechanisms and their biological interpretation.

**Significance Statement**

Despite laboratory susceptibility, gold-standard antibiotics such as vancomycin often fail to clear MRSA bacteremia in vivo. This phenomenon is termed persistent infection and suggests that host–pathogen–antibiotic interactions are essential to methicillin-resistant *Staphylococcus aureus* (MRSA) bacteremia outcomes. Recent studies have identified genetic, transcriptomic, and proteomic determinants of MRSA persistence. However, independently, these determinants provide insufficient mechanistic insight, and it is unclear if they indicate unique or overlapping persistence mechanisms. We use tensor-based decomposition to jointly analyze cytokine and transcriptomic datasets from patients with persistent or resolving MRSA bacteremia. Our results reveal diverging host immune responses that manifest across mechanistic pathways to influence outcomes. These results may guide treatment and future therapeutic discovery by highlighting critical determinants of antibiotic efficacy and protective immunity.

## Introduction

Methicillin-resistant *Staphylococcus aureus* (MRSA) bacteremia is a common and life-threatening infection often arising through community-acquired or healthcare-associated settings (1, 2). These infections are associated with poor outcomes, and up to 30% of appropriate antibiotic regimens fail to resolve bacteremia in vivo despite susceptibility in vitro (3). MRSA bacteremia that resolves upon appropriate antibiotic treatment is termed resolving bacteremia (RB) whereas cases that do not resolve to blood culture-negative status after 5–7 treatment days are termed persistent bacteremia (PB) (4). The limited predictive value of in vitro susceptibility (e.g. minimum inhibitory concentration [MIC] levels) for MRSA bloodstream clearance clinically indicates a need to better understand the host–pathogen determinants shaping antibiotic outcomes in vivo.

Recent progress has been made in identifying determinants of PB vs. RB outcomes in MRSA bacteremia (4–6). Host factors appear to play an important role in MRSA persistence, as patient outcome can be independent of strain susceptibility to vancomycin in vitro (3). Persistence factors are distinct from those associated with MRSA antibiotic resistance, where the organism is refractory to the antibiotic both in vitro and in vivo (7). Thus, advances are necessary to better discern and predict therapeutic outcomes in vivo to prospectively intervene to mitigate persistence. We hypothesize that outcomes are determined by the confluence of immunological responses in an individual host, the infecting MRSA strain, and the specific antibiotic and its use in practice.

To address host responses that contribute to such outcomes, we have previously undertaken broad molecular profiling to measure the molecular differences of MRSA bacteremia response (4–6). These systems-level analyses explored genetic (5), transcriptional, and cytokine (6, 8) correlates of MRSA bacteremia persistence outcomes. However, the extent to which these signatures operate as distinct molecular mechanisms of phenotypic immune response or reflect a shared underlying immune program is yet unclear. Therefore, we approached this problem based on the premise that shared patterns of molecular and cellular responses might improve understanding of the clinical correlates of outcome if they each reflect integrated immunological mechanisms.

Defining molecular and cellular immune signatures can be facilitated by mathematical techniques to identify patterns across large-scale datasets. Matrix and tensor factorization techniques are especially powerful tools for reducing the dimensionality of complex data. Most generally, these methods reduce high-dimensional datasets (data consisting of multiple dimensions, such as measurements, patients, and time) into dimension-specific factor matrices that individually capture patterns across each dimension. These factor matrices individually reveal unforeseen relationships among the diverse datasets and, when recombined, approximate the original measurements. Further, when appropriately matched to the structure of the data, these techniques help to visualize its variation, reduce noise, impute missing values, and reduce dimensionality (9). For data in matrix form, principal components analysis (PCA) and non-negative matrix factorization are two widely applied examples (10). However, when integrating data of higher dimensionality, higher-order generalizations of these methods, tensor factorizations, can be exceedingly useful (9). A particularly important benefit of decomposing data into factor matrices is that it is naturally suited to combining different sources of data, which often derive from diverse biological measurements. Variation along each dimension of data in tensor form is effectively separated by these techniques (11, 12). When integrating two sources of data, each in a matrix or tensor format, coupled matrix-tensor factorization (CMTF) allows detection of shared patterns between datasets of differing dimensionality (11–13). Coupling shared dimensions across datasets provides two distinct benefits; (i) the extent of data reduction is increased by using a common set of patterns across both datasets; and (ii) patterns distinguished in the shared dimension reflect the trends presented in both datasets; thus, their definition is better shaped and meaning integrated. Consequently, retrospective associations or prospective predictions based on these factorized patterns may be improved through more accurate derivation, and interpretation of the resulting patterns may be improved by a more holistic view of the measurements tied to those patterns (11, 14).

In the present study, we applied CMTF to integrate the transcriptional and cytokine responses relative to persistent vs. resolving clinical outcomes in two cohorts of patients with MRSA bacteremia. Data integration enabled the identification of consistent patterns of immunologic response across cytokine and transcriptomic measurements and revealed patterns distinguishing PB from RB outcomes. The combined immunologic patterns explain outcome better than either data type on its own, with correlative accuracy verified in an independent cohort. These associations are shaped by molecular (e.g. cytokine) and cellular (e.g. granulocyte, lymphocyte) differentiation patterns that retain their correlational accuracy when used alone. Overall, the current results demonstrate that robust correlative relationships detected by tensor-based modeling reveal integrative immunological signatures of outcomes in human MRSA bacteremia.

## Results

### A tensor-based strategy for integrating heterogeneous clinical measurements

**Fig. 1.**
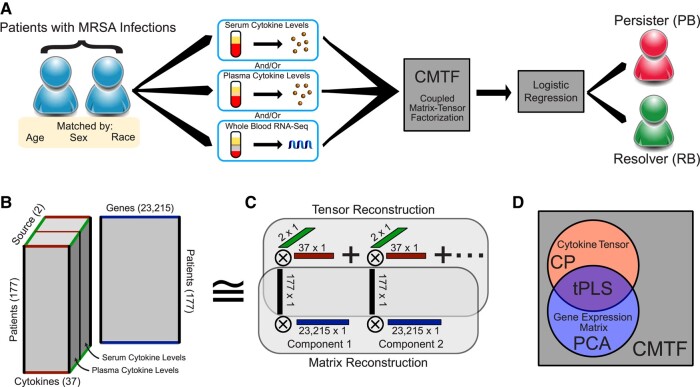
Structured data decomposition integrates clinical measurements with varying degrees of overlap. A) General approach description. Patients with MRSA bacteremia treated with vancomycin had samples collected at admission and then were monitored 5 days post-admission for clearance of MRSA from the bloodstream. Measurements of patient serum cytokine, plasma cytokine, and whole blood transcriptional profiles were assessed. These measurements were then reduced into overall factors describing patterns within the data, which in turn were used to assign disease outcomes, defined as resolving (RB) or persisting (PB) bacteremia. B) Overall structure of the data. Cytokine measurements from either plasma or serum can be arranged in a 3D tensor, wherein each dimension indicates patient, cytokine, or sample source, respectively. In parallel, gene expression measurements are aligned with cytokine measurements by virtue of sharing patients. C) Data reduction is performed by identifying additively separable components represented by the outer product of vectors along each dimension. The patient factors are shared across both the tensor and matrix reconstruction. D) Venn diagram of the variance explained by each factorization method. Canonical polyadic (CP) decomposition can explain the variation present within the cytokines tensor, or principal component analysis (PCA) could be used to reduce the gene expression matrix (9). Tensor partial least squares regression (tPLS) allows one to explain the shared variation between the matrix and tensor (15, 16). In contrast, here we wish to explain the total variation across both the tensor and matrix. This is accomplished with CMTF (11–13).

To identify common patterns across cytokine and RNA-seq measurements, we first sought to optimally organize the multiple datasets. Three types of measurements were collected—plasma cytokines, serum cytokines, and RNA-seq from whole blood samples (Fig. 1A). Known differences exist in cytokine measurements between plasma and serum; however, certain shared variation across patients is expected and thus the cytokine measurements across serum and plasma sources should have some matching correspondence. Therefore, while not every patient had every type of measurement, the study contained two types of cytokine measurements, aligned across cytokines and patients, coupled with RNA-seq measurements that shared a common patient dimension.

In situations where measurements can be aligned along two or more dimensions, the data can be structured into tensor form. A generalization of a matrix, which is a two-mode tensor, tensors most often refer to organized arrays with three or more dimensions (modes); in the three-mode form, tensors are structured as a cube of measurements. The cytokine data was structured into a 3-mode tensor, with patient, cytokine, and cytokine source (serum or plasma) axes (Fig. 1B). This tensor was paired with the RNA-seq measurements in a matrix containing the shared patient axis and a separate gene axis. To integrate both data types, we used CMTF (11–13). This method solves for an optimal low-rank approximation of both datasets while keeping the coupled dimension factors (in this case, patients) shared (Fig. 1C). As CMTF maximizes the variance explained across both datasets, it is better suited here than other approaches that maximize explanation of other subsets of variance, including canonical polyadic (CP) decomposition for just the cytokine tensor, PCA for just the RNA-seq matrix, or partial least squares regression in tensor form (tPLS) to exclusively examine the shared variance (Fig. 1D).

### Tuning dimensionality reduction for accurate correlations with MRSA bacteremia outcomes

**Fig. 2.**
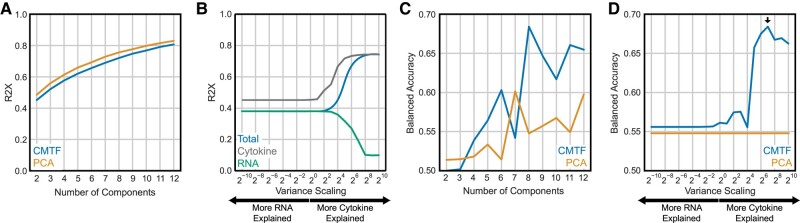
CMTF parameter tuning to correlate bacteremia outcome. A) Number of components used in CMTF and PCA decomposition vs. the percent variance reconstructed (R2X). B) Percent variance explained upon reconstruction (R2X) of the entire data, RNA, or cytokine measurements with varying scaling between the two datasets. C, D) Balanced accuracy in assigning bacteremia outcomes with varying number of components (C) and scaling (D).

Dimensionality reduction via CMTF introduces two method parameters that influence the resulting decomposition. First, decomposition can be performed using a varying number of components. A small number of components effectively explained the data variation, with eight components capturing >70% of the total variance while reducing the data to 16% of its original size (2,192 factor values vs. 14,132 nonmissing measurements; Fig. 2A). To compare against other dimensionality reduction techniques, PCA was applied to all measurements concatenated in a single matrix. We found that CMTF essentially captured the same amount of data variation compared to PCA at an equivalent number of components. Second, as CMTF aims to maximize the total variance of both datasets explained, the relative numerical scale between cytokine and RNA-seq measurements affects the goodness of fit for each individual dataset; CMTF prioritizes explaining patterns in the dataset with the larger scale. To explore the effect of this scaling, we tested a variety of scales; the resulting factors were responsive to the relative scale of each data type (Fig. 2B). Note that, when the emphasis on one dataset is greatly increased, the total R2X overlaps with the over-emphasized dataset; this is expected, as most of the variance in the two datasets is contained in the over-emphasized measurements.

We chose to identify reduced patterns that were optimally able to correctly assign PB vs. RB outcomes. To do so, clinical variables were correlated with outcome by logistic regression. Assignment accuracy was quantified using 10-fold cross-validation. Briefly, 10% of the patients were left out from the logistic regression model, and the remaining 90% were used to learn the relationship between each component and outcome. Next, the logistic regression model was used to assign outcomes for patients held out of the model training. This process was repeated until every patient was categorized with respect to PB vs. RB based on cytokine and transcriptome profiles. Reported accuracies are the balanced accuracy scores observed over this cross-validation process. Using this process while varying the settings within CMTF, a peak correlation performance is observed at eight components (Fig. 2C) and when the cytokine data were scaled to have a total variance 128 times larger than the RNA-seq data (Fig. 2D). CMTF's peak performance exceeded that of PCA over a wide range of scaling values and component numbers (Fig. 2C and D).

### Coupled factors improve the accuracy of discerning MRSA bacteremia outcomes

**Fig. 3.**
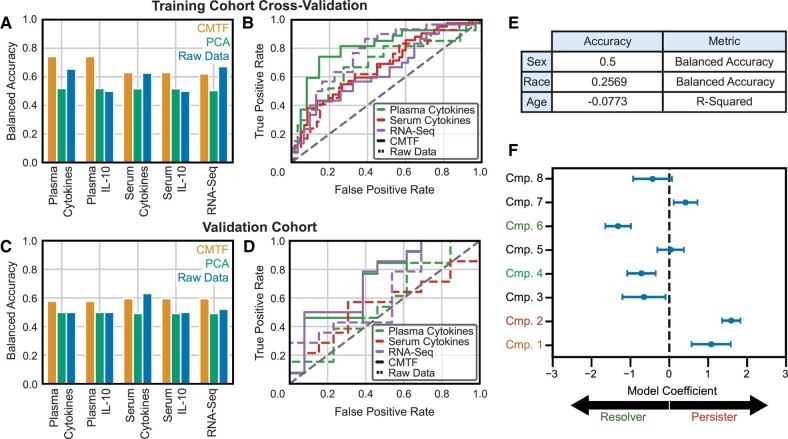
CMTF improves assignment accuracy of persistent MRSA bacteremia. A) Balanced accuracy in RB/PB assignment from models trained with CMTF components, PCA components, and raw data sources. Accuracy is evaluated using 10-fold cross-validation over the training cohort. Model accuracies are evaluated over subsets of patients dependent on their available measurements; the CMTF model considers all available measurements for all patients. B) Receiver operating characteristic curves for the models depicted in (A). C) Balanced accuracy in RB/PB assignment from models trained with CMTF, PCA, or the raw data sources. Model accuracy is evaluated against a masked validation cohort following training against the training cohort. D) Receiver operating characteristic curves for models depicted in (C). E) CMTF model performance in assigning auxiliary demographics. Four unique races were reported, so random chance for the “Race” prediction is a balanced accuracy of 0.25. Random chance for all other categorical variables is 0.50. F) Model coefficients assigned to each CMTF component. Dots and error bars depict the bootstrapping means and standard deviations of model coefficients, respectively.

We next sought to evaluate the extent to which CMTF-derived patterns could accurately distinguish MRSA PB vs. RB outcomes. We built a regularized logistic regression classifier to assign persistence outcome from the CMTF components and compared its performance to those built with a single data source or with an eight-component PCA scores matrix derived from all measurements concatenated into a single matrix (Fig. 3A and B). As elevated IL-10 is associated with PB (4, 5), logistic regression models were also constructed to assign persistence outcome from IL-10 measurements alone. Consistently, CMTF-derived factors more accurately differentiated PB vs. RB outcomes than matrix-based models (Fig. 3A). Note that, because each data source was not available for all patients, comparisons were made using the subset of patients having the respective data measurements; the CMTF model, however, is the same across patient subsets and considered all available measurements for all patients. As further validation, model performances were compared for a separate cohort that remained blinded during the model assembly process. While there was an expected decrease in accuracy overall, CMTF-derived factors were again more effective at differentiating PB vs. RB outcomes (Fig. 3C and D). Thus, data integration improved the accuracy of PB assignment (Fig. 3A).

We also sought to evaluate any relationships between the CMTF-derived components and patient demographic characteristics, specifically biological sex, age, and race (Fig. 3E). MRSA susceptibility is known to vary with each of these parameters, and so we surmised that persistence may be influenced by factors that also vary with these characteristics (7). However, neither sex, age, nor race correlated better than chance, and there were no significant associations of the molecular components studied with these demographic features (Fig. 3E). This lack of association between components and such clinical characteristics may be attributed to our patients being matched on basis of sex, age, and race; regardless, the poor associations of the components with these clinical characteristics indicates their inclusion would not improve recognition of persistence-associated molecular patterns in this study.

A benefit of the logistic regression model is its ease of interpretation. As part of the fitting process, the logistic regression model assigns a coefficient to each CMTF component (Fig. 3F). These coefficients indicate the relative impact of each CMTF component in MRSA PB vs. RB outcomes; more influential components have coefficients of greater magnitude. Additionally, the sign of the coefficient informs the directionality of the association; positive coefficients indicate an association with PB outcomes, while negative coefficients associate with RB outcomes. Uncertainty in each coefficient was quantified by bootstrapping the patient factors produced via CMTF (17). CMTF components 1 and 2 strongly associated with the PB outcome while components 4 and 6 strongly associated with the RB outcome (Fig. 3F).

To provide a comparison baseline, alternative matrix-based models were built to predict MRSA bacteremia persistence outcome from a flattened version of our dataset that concatenated all available measurements into a matrix (Fig. S3). We applied an eight-component PCA to this concatenated matrix to reduce dimensionality and impute missing values; logistic regression and support vector machine classification models were used to predict persistence outcome from the resulting PCA scores matrix and PCA-imputed concatenated matrix. CMTF consistently outperformed the matrix-based models.

### Coupled factors reveal conserved immunological responses in MRSA bacteremia

**Fig. 4.**
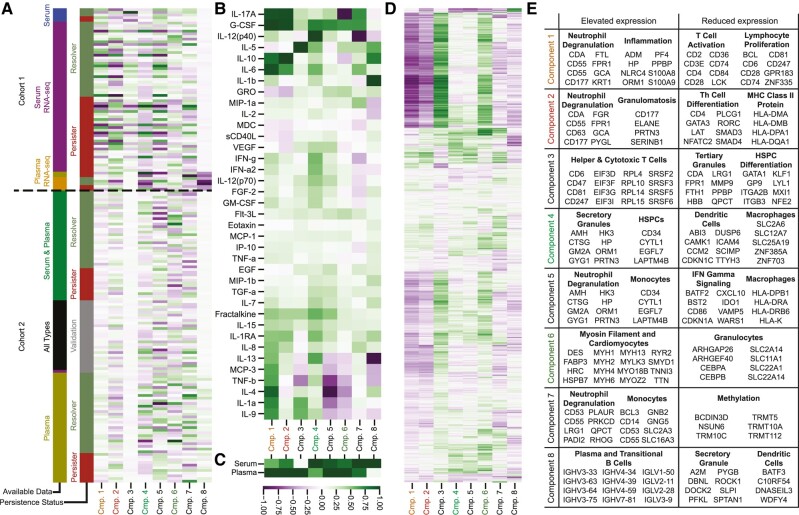
CMTF components identify conserved patterns of MRSA immunologic response. A) Component associations with each patient. B) Component associations with each measured cytokine. C) Component associations with the two cytokine sources: plasma and serum. D) Component associations with the RNA-seq measurements. E) Enrichment analysis for genes associated with elevated and reduced expression for each component. Selected genes are representative of their corresponding gene set.

We plotted the composition of each CMTF component against the four factor dimensions (patient, cytokine, serum vs. plasma, and RNA expression) to evaluate the relative biological significance of each component (Fig. 4A–D). Factor components were scaled to have a range of −1 and 1. Next, gene enrichment analysis was performed on the genes most positively and negatively associated with each component to identify enriched transcriptomic processes (Fig. 4E).

These factor matrices can be interpreted in two primary ways. To interpret the biological significance of a particular CMTF component, one can evaluate its composition across the factor matrices. For instance, component 1 corresponded to upregulation of IL-17A, G-CSF, IL-10, IL-6, and IL-4 (Fig. 4B) in plasma cytokine measurements (Fig. 4C). Component 1 also correlated with the greater expression of genes associated with neutrophil degranulation and inflammation and reduced expression of genes associated with T cell activation and lymphocyte proliferation (Fig. 4D and E). In parallel, one can also compare the differences between components by examining each individual factor matrix. Components 1 and 2, for example, were mostly similar in their RNA-seq gene associations (Fig. 4D) and serum and plasma cytokine sources (Fig. 4C) but were distinct in their association with cytokines like IL-4 (Fig. 4B).

Components 1, 2, 4, and 6 were important correlates of persistent MRSA bacteremia outcome (Fig. 3F). Component 2, which correlated with PB outcomes, associated with upregulation of plasma IL-17A, G-CSF, IL-10, and IL-6 cytokine measurements alongside greater expression of gene signatures associating with neutrophil degranulation and granulomatosis and lesser expression of CD4+ T-helper cell differentiation genes (Fig. 4). Component 4, which associated with RB outcomes, associated with upregulation of serum IL-12(p40) cytokine measurements, increased expression of genes associated with granulocyte granules and hematopoietic stem cells (HSPCs), and decreased expression of genes associated with dendritic cells and macrophages. Component 6, which also associated with RB outcomes, corresponded to downregulation of IL-17A and upregulation of G-CSF across serum and plasma cytokine measurements alongside greater expression of genes associated with cardiomyocytes and lesser expression of genes linked to granulocytes.

The remaining CMTF components, 3, 5, 7, and 8, identified biological processes across RNA-seq and cytokine measurements that did not strongly associate with persistent MRSA bacteremia outcome. Of these components, component 3 associated strongly with IL-5 upregulation and with increased expression of helper (CD4+) and cytotoxic T cell (CD8+) genes alongside decreased expression of genes associated with tertiary granules and HSPC differentiation. Component 5 corresponded with the downregulation of IL-4 and TNFβ, elevated expression of neutrophil degranulation and monocyte genes, and decreased expression of genes associated with IFNγ signaling and macrophages. Component 7, like component 5, associated with greater expression of neutrophil degranulation and monocyte genes, but was also associated with lesser expression of methylation gene signatures and IL-12(p40). Finally, component 8 corresponded to upregulation of IL-1b and downregulation of IL-13, greater expression of genes associated with plasma and transitional B cells, and reduced expression of genes associated with secretory granules and dendritic cells.

Notably, components 3 and 8 appear to be primarily influenced by batch effects. The magnitudes of these components were distinct between cohorts, suggesting that these components identified signals with cohort-to-cohort differences (Fig. 4A). Dimensionality reduction techniques, including tensor factorization, are routinely used for correcting batch effects (18, 19), and these batch-associated components help to capture patterns associated with batch effects so that the remaining components may better associate with persistence-related mechanisms.

### A reduced model reveals heterogeneity in persistent MRSA bacteremia outcomes

**Fig. 5.**
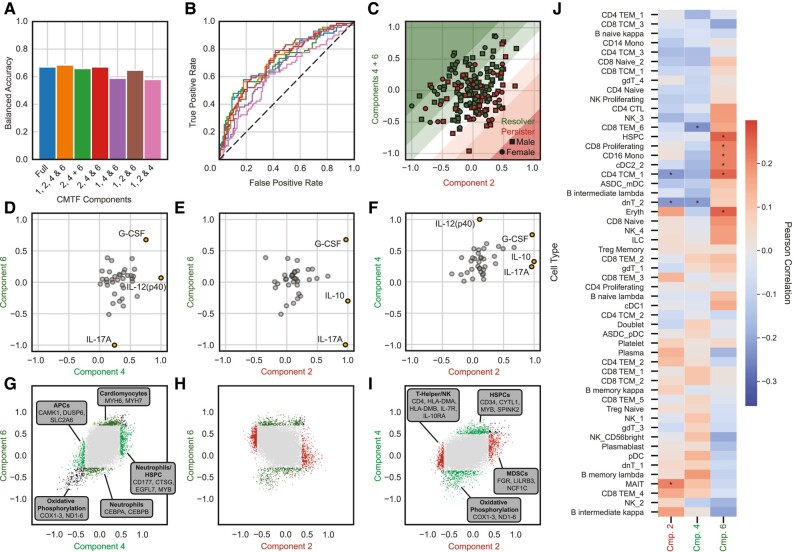
A reduced model visualizes heterogeneity in persistent MRSA bacteremia outcomes. A) Balanced accuracy scores of support vector machine classification models trained with subsets of CMTF components. Accuracy is evaluated using 10-fold cross-validation over all patients. B) Receiver operating characteristic curves for models depicted in (A). C) Patient factor values for CMTF component 2 vs. the sum of components 4 and 6. Red and green shading indicate persister- and resolver-predicted patients, respectively. D–F) Pairwise comparisons between cytokine factor values for CMTF components 2, 4, and 6. Cytokine factors with large absolute differences between components are highlighted in orange. G–I) Pairwise comparison between RNA expression factors for CMTF components 2, 4, and 6. The top 500 genes most-associated with reduced and elevated expression are highlighted in red (component 2), cyan (component 4), and green (component 6). Genes associated with reduced or elevated expression in both components are shown in black. J) Component correlations to immune cell quantities inferred via CIBERSORTx. Asterisks indicate significant correlation (*P* < 0.05) between immune cell and component.

Given that only a subset of components strongly associated with MRSA persistence, we next sought to test whether a reduced model using few components could be equally associative with persistence as the full model. To address this, we built logistic regression models that used subsets of components 1, 2, 4, and 6 to predict persistence outcome. We compared these reduced models to a logistic regression model that used all eight CMTF components. In contrast to our earlier analysis, we included all 177 patients in this analysis. A reduced model trained with only component 2 (upregulation of IL-17A, G-CSF, IL-10, IL-6, neutrophil degranulation/granulomatosis, and downregulation of T-helper cell differentiation) and the sum of components 4 (upregulation of IL-12(p40), granulocyte granules, HSPCs, and downregulation of dendritic cells and macrophages) and 6 (downregulation of IL-17A, granulocytes and upregulation of cardiomyocytes) had equal classification accuracy to the model built with all components (Fig. 5A and B), supporting the notion that components 2, 4, and 6 are informative of bacteremia persistence outcome on their own.

With the reduced model indicating the independent value of components 2, 4, and 6, we then plotted the patients within this reduced space (Fig. 5C). Here, a clear subset of patients with RB is found in the top-left that the model correctly identifies as RB. This relationship indicates that higher combined component 4 and 6 values coupled with lower component 2 values strongly associated with RB; that is, increases in RB-associated components 4 and 6 in conjunction with decreases in PB-associated component 2 associated with RB.

To examine the biological distinctions between components 2, 4, and 6, we plotted the cytokine (Fig. 5D–F) and RNA-seq (Fig. 5G–I) factor values of components 2, 4, and 6 against each other. G-CSF is positively associated with each component. The RB-associated components 4 and 6, however, were unique in their associations to other cytokines; component 4 associated positively with IL-12(p40) while component 6 associated negatively with IL-17A. The PB-associated component 2, conversely, was unique from the RB-associated components in its positive correlations to IL-17A and IL-10. With regards to transcriptomic associations, RB-associated components 4 and 6 had similar RNA-seq associations as both associated with reduced expression of genes involved in oxidative phosphorylation. Examination of genes unique to components 4 and 6, however, found that component 4 associated with greater expression of neutrophil and HSPC-associated genes alongside reduced expression of macrophage and dendritic cell genes while component 6 associated with greater expression of cardiomyocyte genes and lesser expression of neutrophil transcriptomic signatures. Components 2 and 6 showed minimal correlation in their transcriptomic associations but, surprisingly, components 2 and 4 demonstrated some similarities in their RNA-seq associations as both components 2 and 4 associated with increased expression of neutrophil-associated genes. Component 2, however, also involved elevated expression of myeloid-derived suppressor cell (MDSC) genes and reduced expression of helper T and NK cell signatures while component 4 associated with reduced expression of oxidative phosphorylation genes and greater expression of HSPC-associated gene patterns.

To further validate these findings, we applied CIBERSORTx to the RNA-seq measurements to infer immune cell quantities (20). To associate these immune cell quantities with our components, CMTF patient factors were correlated with the CIBERSORTx-inferred immune cell quantities (Fig. 5J). Component 2 correlated with reduced CD4 TCM1 and double-negative T cells along with increased mucosal-invariant T cells. Component 4, like component 2, involved reduced double-negative T cells but also correlated with reduced CD8 TEM6 cells. Component 6, finally, correlated with increased HSPC, CD8 proliferating, CD16 monocyte, type 2 dendritic cell, CD4 TCM1, and erythrocyte populations.

Finally, to assess whether the transcriptomic signatures would have been revealed through simpler analysis, we compared the transcriptomic factors of components 2, 4, and 6 against the genes found differentially expressed with PB vs. RB status (Fig. S5) (21). Gene enrichment analyses of these differentially expressed genes found significantly elevated expression of signatures associated with granulocytes, dendritic cells, macrophages, and monocytes while genes associated with T-helper cells (including Th2 and Th17), B cells (memory, naïve, and double-negative), NK cells, and cytotoxic T cells demonstrated significantly reduced expression. While many of the immune cell signatures were also found in components 2, 4, and 6, these components were unique from the differential expression analysis as each component highlighted different subsets of immune cells.

## Discussion

**Fig. 6.**
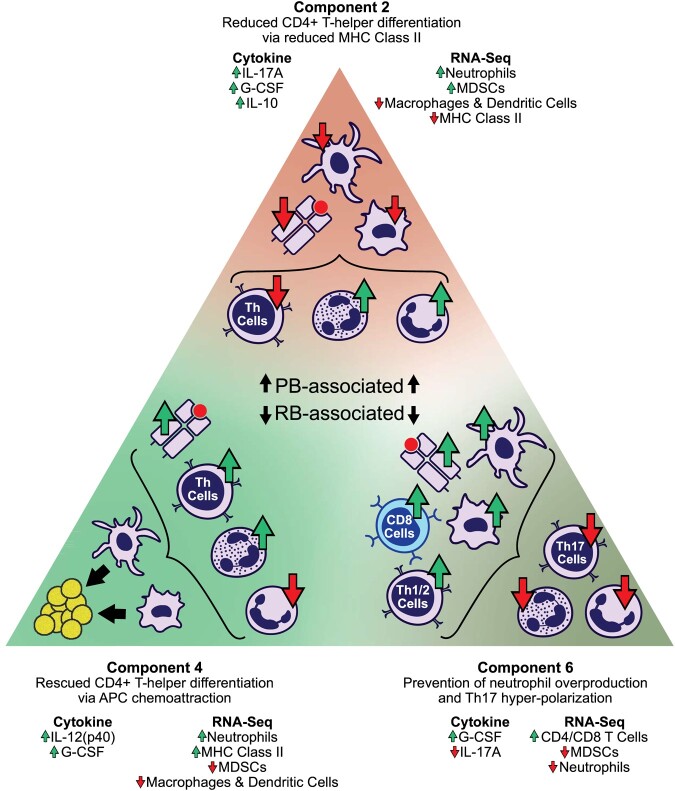
CMTF suggests granulocyte maturation, antigen-presenting cell function, and lymphocyte differentiation are determinants in MRSA bacteremia persistence. Overview of hypothesized immunological determinants of MRSA bacteremia outcomes from CMTF. Each axis reflects patients’ positive or negative association with that component pattern. For instance, PB outcomes are associated with positive weighting along component 2 alongside negative weights along components 4 and 6.

Here, results demonstrate that CMTF can improve the understanding of host immune responses correlated with outcomes in human MRSA bacteremia. This insight is gained by characterizing the host immune response through the integration of multiple data types. We find that CMTF captures over 70% of the variation observed across clinical RNA expression and cytokine measurements in just 8 components, that these components strongly associate with MRSA bacteremia persistence vs. resolution (Fig. 2). Furthermore, these components more accurately predict persistence outcome than standard matrix-based and single-datatype models (Fig. 3). Our prediction model indicates each component's association to persistence, while CMTF supplies information about how each component relates to individual measurements. Consequently, we can plausibly interpret these components to better understand the underlying immunologic mechanisms (Fig. 4). We find a subset of components are equally associated with persistence as all eight components (Fig. 5). Interestingly, examination of this component subset affords more precise delineation of immune heterogeneity in immune responses to MRSA bacteremia. This observed heterogeneity in immune responses and key immunological signatures associated with PB and RB outcomes are highlighted in Fig. 6.

In integrating the serum and plasma cytokine with whole blood RNA-seq measurements, we found that no one data type was consistently most predictive of outcome on its own (Fig. 3). Certain data types, such as the plasma cytokines, showed greatest predictive accuracy within the training cohort, but this did not extend to the validation cohort. Thus, one benefit of data integration may be to leverage a larger sampling of individuals, resulting in more robust associations. Additionally, while previous studies have found associations between sex, race, and age with persistent MRSA bacteremia (7), the CMTF components were not associative with these auxiliary demographics. It is therefore conceivable that the molecular and cellular patterns identified in the present study associated with persistence through means independent of demography.

The observed differences in cytokine profiles typically generated by distinct CD4+ T-helper cell polarization in response to infection implies T cell responses are integral to shaping outcomes in MRSA bacteremia. This concept is consistent with findings regarding epigenetic correlates of such outcomes (4). In the present study, further interrogation of the cytokine and gene expression factors relevant to each component also support and extend existing literature in MRSA persistence mechanisms. For example, component 2 associated strongly with PB and with G-CSF, IL-17A, and IL-10 upregulation. Elevated levels of G-CSF and IL-17A are linked to increased quantitative and potent neutrophil responses in staphylococcal infection (4, 22), suggesting component 2 may correspond to a neutrophil dysfunction. Indeed, MRSA has well-documented methods of avoiding neutrophil recognition (23), and recent reports have associated elevated immature granulocyte formation in response to MRSA infection with PB (4). Alternatively, elevation in neutrophil response could also indicate elevated bacterial burden; MRSA elicits neutrophil formation and recruitment, and proliferative neutrophil response may correspond to heightened infection severity rather than neutrophil dysfunction. However, in PB and other conditions in which the immune response is nonprotective, neutrophil count may not correspond with pathogen burden. It follows that upregulation of IL-10 and IL-17A have been independently linked with persistent MRSA bacteremia (4, 5, 24–27). Indeed, the present finding that component 2 associated with upregulation of both IL-10 and IL-17A in PB suggests the two cytokines share an underlying mechanism. Furthermore, component 2 also associated with elevated expression of neutrophil gene signatures and reduced expression of genes associated with MHC class II and CD4+ T-helper cell differentiation. This pattern of results implies that the upregulation of IL-10 and IL-17A with elevated neutrophil populations in context of reduced CD4+ T-helper differentiation potentially follows deviations from protective MHC class II antigen presentation.

Intriguingly, component 4 exhibited similar cytokine and transcriptional signatures to component 2 but an opposite association with persistence outcome. Both components strongly associated with G-CSF upregulation, which promotes neutrophil production, maturation, and distribution (28) alongside transcriptomic signatures associated with neutrophils. These findings suggest both components corresponded to increases in neutrophil production. Unlike component 2, however, component 4 associated with elevated IL-12 (p40) that typically associates with antigen-presenting cell (APC) response early in the course of infection, and subsequent downregulation of IL-10 (29–31). Alternatively, elevated IL-12 (p40) could indicate an elevation in IL-23—a pro-inflammatory cytokine with an IL-12 (p40) subunit that is produced by APCs (32). However, in context of component 4's transcriptomic reduction in APC response and weak association to IL-17A, a downstream target amplified by IL-23 expression, it is unlikely this component corresponded to elevated IL-23. In comparing transcriptomic signatures between components 2 and 4, we found further reduction of immunosuppressive signals in component 4. While component 4's transcriptomic factor associated with reduced dendritic cell and macrophage signatures, this component did not associate with reduced CD4+ T-helper differentiation, NK cell response, or MHC class II antigen presentation. It is plausible that this pattern is potentially the result of elevated IL-12 (p40), a chemoattractant for both dendritic cells and macrophages (33, 34). Hypothetically, improved macrophage and dendritic cell infiltration may compensate for reduced MHC class II antigen presentation by other cell subsets. This rescue of MHC class II function may, in turn, be responsible for improved CD4+ T-helper differentiation (35) or polarization. Furthermore, the reductions in dendritic cell and macrophage transcriptomic signatures from whole blood supports a plausible hypothesis that APCs migrate into infected tissues. These findings are consistent with previous reports that found macrophages are integral to APC protective immunity against MRSA infections (6, 36–38). The present findings offer previously unseen insights, however, suggesting that improved trafficking and antigen presentation by macrophages may facilitate antigen presentation resulting in CD4+ T-helper cell polarization to drive protective immune programs.

Like component 4, component 6 was associated with RB; unlike components 2 and 4, however, component 6 comprises pathways involved with regulation of neutrophil function. For example, IL-17A is a pro-inflammatory Th17 cytokine that promotes neutrophil-mediated immune responses (39). Reduced expression of IL-17A, coupled with reduced expression of granulocyte gene signatures, suggests component 6 reflects reduced granulocyte response. We note that the cause of this reduced granulocyte response is uncertain and could reflect a paradoxical relationship in which infection severity and granulocyte response are decoupled. Regardless, comparing transcriptomes between components 4 and 6 revealed commonality among reduced expression of gene signatures associated with oxidative phosphorylation. This pattern could indicate reduction in reactive oxygen species (ROS) formation among immature neutrophils and may suggest a separate mechanism to mitigate suppression of CD4+ T cell polarization. Specifically, ROS induce myeloid-derived suppressor cells (MDSC) that suppress the immune response (40, 41). Hence, impairment of ROS and ensuing MDSC response may avoid suppression of CD4+ response and support RB outcomes. Supporting this interpretation, component 6 involved no association to MDSCs and its CIBERSORTx correlations indicated upregulation of CD4+ and CD8+ T cell proliferation as well as dendritic cell functions, suggesting an immunologic program to promote CD4+ T and dendritic cells interactions that were suppressed in component 2. A complementary hypothesis is that reduced oxidative metabolism could reflect attenuation of mitochondrial ROS and antiapoptotic signaling in neutrophils (42, 43). This possibility is supported by neutropenia often observed in PB outcomes. Collectively, the noted differences among CMTF components 2, 4, and 6 offer unforeseen insights into immune cell generation, differentiation, and trafficking that collectively shape outcomes in MRSA bacteremia.

We acknowledge that this investigation has limitations. First, while this study investigates a well-characterized cohort, it is a moderately sized sample of 177 patients collected from a single center. Second, while our validation cohort was blinded to investigators throughout the study, it was a subset of a patient cohort used in model training. Thus, the current findings lay an important foundation for future validation studies in demographically and geographically independent patient cohorts. As our dataset consisted of patients across two cohorts, however, we believe that these findings are likely to reflect generalizable MRSA bacteremia persistence mechanisms. Third, while the current study focused on host response contributions to outcomes, MRSA virulence mechanisms are known to vary with the route-of-acquisition and MRSA strain (44, 45). Hence, evaluation of such variables in ongoing studies may reveal further insights. Fourth, cytokine and transcriptomic measurements were collected at a single timepoint, limiting study of longitudinal relationships of the observed mechanisms. For instance, while our findings linked IL-10 and IL-17A upregulation into one PB-associated process, it is uncertain if this mechanism is driven by IL-10, or if IL-10 upregulation is a regulatory response to IL-17A driven inflammation. Similarly, while our findings link transcriptomic reductions in T-helper differentiation and MHC class II proteins with PB, it is uncertain if reduction in T-helper differentiation is a cause or effect of MHC class II protein reduction. Finally, this study considered only patients with MRSA bacteremia treated with a vancomycin therapy over a 5-day course. While this therapeutic regimen reflects the most common real-world clinical scenario, whether immunological signatures found translate to other antibiotic regimens is unknown. Similarly, it is not yet known whether these immunological signatures are unique to MRSA bacteremia, extendable to MRSA infections in other tissue compartments (e.g. skin infection or organ abscess) or generalizable across other pathogens.

In summary, these results find that an integrated view of cytokine and RNA expression obtained using CMTF may improve understanding of the immune response shaping outcomes of MRSA bacteremia. Integrating data types to define molecular patterns of immunologic response provides dual benefits. Firstly, interpretation of the resulting patterns is made easier through a broader view of the involved molecular factors. Additionally, immune response patterns, especially with data reduction in tensor form, are more precisely defined through more unified dimensionality reduction. Importantly, immune mechanisms associated with outcomes in MRSA bacteremia manifest over multiple biological modalities as revealed through tensor factorization methods that may be missed by conventional modeling methods. This is a key benefit of tensor factorization methods; opposed to matrix-based methods, like PCA, that can conflate cross-modality patterns by concatenating measurements into a single matrix, CMTF excels in capturing and revealing MRSA bacteremia persistence mechanisms through dimension-specific component associations. Thus, these results demonstrate the importance of multiomics data profiling and their integration in characterizing the human immune response, with coupled tensor factorization as a powerful tool for pattern discovery and plausible data interpretation.

## Materials and methods

### Patients and sample collection

This study was conducted in accordance with Good Clinical Practice and Human Subjects Research as approved by the Duke University Medical Center (DUMC) Institutional Review Board. Staphylococcus bacteremia (SAB) patients were evaluated and consented for enrollment in the *S. aureus* Bacteremia Group (SABG) repository at DUMC. All patient samples were deidentified prior to use in this study. This case-controlled study consisted of 177 SAB patients (71 PB and 106 RB) propensity matched by sex, race, age, hemodialysis status, type I diabetes, and presence of an implantable device. These 177 patients consisted of SAB patients from two cohorts, with 68 patients from cohort 1 (34 PB and 34 RB) and 109 patients from cohort 2 (37 PB and 72 RB). Both patient cohorts were collected from the same study group and underwent the same inclusion criteria and data collection procedures outlined below; the only difference between cohorts is that cohort 2 samples were analyzed later. Of the 109 patients in cohort 2, 27 patients (14 PB and 13 RB) were removed and blinded to the investigators as a validation cohort. Details of clinical characteristics of study cohort are presented in Table 1. Of the 177 patients, 129 had serum cytokine measurements, 115 had plasma cytokine measurements, and 88 had RNA-seq measurements. Of the 27 validation cohort patients, 26 had all three measurement types available; the remaining validation cohort patient had only serum cytokine and RNA-seq measurements. SAB cases were evaluated and consented for enrollment in the SABG biorepository at DUMC.

**Table 1.** Demographic and clinical characteristics of the patients with resolving and persistent bacteremia.

	Overall (*N* = 177)	Resolving bacteremia (*N* = 108)	Persistent bacteremia (*N* = 69)	*P*-value
Demographics				
Age, mean (median), years	61 (63)	62 (63)	63 (64)	0.38
Sex				0.42
Male	110 (62.1%)	66 (61.1%)	44 (63.8%)	
Female	67 (37.8%)	42 (38.9%)	25 (36.2%)	
Race				0.36
Black	67 (37.9%)	39 (36.1%)	28 (40.6%)	
White	106 (59.9%)	68 (63.0%)	38 (55.1%)	
Other	4 (2.3%)	1 (0.9%)	3 (4.3%)	
Route of infection				0.03
Hospital acquired	23 (13.0%)	19 (17.6%)	4 (5.8%)	
Community acquired, healthcare associated	122 (68.9%)	67 (62.0%)	55 (79.7%)	
Community acquired, nonhealthcare associated	32 (18.1%)	22 (20.4%)	10 (14.5%)	
Initial source of bacteremia				NA
Skin/soft tissue/osteoarticular	51 (28.8%)	36 (33.3%)	15 (21.7%)	
Endovascular^a^	50 (28.2%)	28 (25.9%)	22 (31.9%)	
Pulmonary^b^	17 (9.6%)	12 (11.1%)	5 (7.3%)	
GI/GU	7 (4.0%)	5 (4.6%)	2 (2.9%)	
Unknown/other	49 (27.7%)	27 (25.0%)	22 (31.9%)	
Metastatic infection	91 (51.4%)	41 (38.0%)	50 (72.5%)	<0.01
Apache II, mean (range)	16.6 (2–50)	16.1 (2–50)	17.4 (6–36)	0.90
Length of stay (days)				<0.01
<9	30 (16.9%)	30 (27.8%)	0 (0.0%)	
9–14	49 (27.7%)	33 (30.6%)	16 (23.2%)	
15–20	39 (22.0%)	16 (14.8%)	23 (33.3%)	
>20	59 (33.3%)	29 (26.9%)	30 (43.5%)	
Procedures				
Surgical removal of foreign device	57 (32.2%)	22 (20.4%)	35 (50.7%)	<0.01
Surgical debridement	36 (20.3%)	20 (18.5%)	16 (23.2%)	0.37
Surgical insertion of foreign device	18 (10.2%)	8 (7.4%)	10 (14.5%)	0.13
Abscess drainage	25 (14.1%)	11(10.2%)	14 (20.3%)	0.07
Line removal	18 (10.2%)	14 (13.0%)	4 (5.8%)	
Other	54 (30.5%)	30 (27.8%)	24 (34.8%)	0.31
Comorbidities				
Neoplasm	28 (15.8%)	24 (22.2%)	4 (5.8%)	<0.01
Diabetes mellitus	85 (48.0%)	47 (43.5%)	38 (55.1%)	0.10
Dialysis dependence	37 (20.9%)	16 (14.8%)	21 (30.4%)	0.01
HIV	4 (2.3%)	2 (1.9%)	2 (2.9%)	0.51
Organ transplant recipient	15(8.5%)	10 (9.3%)	5 (7.2%)	0.43
Intravenous drug use	9 (5.1%)	6 (5.6%)	3 (4.4%)	
Steroid use in previous 30 days	44 (24.9%)	30 (28.0%)	14 (20.3%)	
Surgery in previous 30 days	44 (24.9%)	29 (26.9%)	15 (21.7%)	
Outcome, 90 days				0.15
Cure	139 (78.5%)	90 (83.3%)	49 (71.0%)	
Recurrent SA infection	12 (6.8%)	7 (6.5%)	5 (7.3%)	
Death due to SA infection	17 (9.6%)	6 (5.6%)	11 (15.9%)	
Death due to other causes	6 (3.4%)	3 (2.8%)	3 (4.4%)	
Unknown/other^c^	3 (1.7%)	2 (1.9%)	1 (1.4%)	

^a^Endovascular source includes catheters, LVADs, pacemaker/defibrillator, and gortex graft source of infection.

^b^Pulmonary source of infection includes pneumonia and empyema.

^c^Outcome, 90 days, unknown/other includes discharge to hospice, lost to follow-up, and missing.

Plasma and/or sera and whole blood Paxgene samples were collected at time of diagnosis of MRSA infection and stored in the SABG biorepository. Cases for the current study were carefully selected based on the following inclusion criteria: laboratory confirmed MRSA bacteremia; received appropriate vancomycin therapy; enrolled in the SABG study between 2007 and 2017 (to ensure contemporary medical practices) and had available serum or plasma samples. PB was defined as patients had continuous MRSA positive blood cultures for at least 5 days after vancomycin antibiotic treatment (5); RB patients were positive for MRSA in initial blood cultures but then were negative in subsequent cultures.

### Molecular analysis

#### Luminex-based cytokine measurement

Human 38-plex magnetic cytokine/chemokine kits (EMD Millipore, HCYTMAG-60K-PX38) were used per manufacturer's instructions. The panel includes IL-1RA, IL-10, IL-1ɑ, IL- 1β, IL-6, IFN-ɑ2, TNF-β, TNF-ɑ, sCD40L, IL-12p40, IFN-γ, IL-12/IL-12p70, IL-4, IL-5, IL-13, IL-9, IL-17A, GRO/CXCL1, IL-8/CXCL8, eotaxin/CCL11, MDC/CCL22, fractalkine/CX3CL1, IP-10/CXCL10, MCP-1/CCL2, MCP-3/CCL7, MIP-1ɑ/CCL3, MIP-1β/CCL4, IL-2, IL-7, IL-15, GM-CSF, Flt-3L/CD135, G-CSF, IL-3, EGF, FGF-2, TGF-β, and VEGF. Fluorescence was quantified using a Luminex 200TM instrument. Cytokine/chemokine concentrations were calculated using Milliplex Analyst software version 4.2 (EMD Millipore). Luminex assay and analysis were performed by the UCLA Immune Assessment Core.

#### RNA sequencing, mapping, quantification, and quality control

Total RNA was isolated with Qiagen RNA Blood kit, and quality control was performed with Nanodrop 8000 and Agilent Bioanalyzer 2100. Globin RNA was removed with Life Technologies GLOBINCLEAR (human) kit. Libraries for RNA-Seq were prepared with KAPA Stranded mRNA-Seq Kit. The workflow consists of mRNA enrichment, cDNA generation, and end repair to generate blunt ends, A-tailing, adaptor ligation, and PCR amplification. Different adaptors were used for multiplexing samples in one lane. Sequencing was performed on Illumina Hiseq3000 for a single 50M-read run. Each sample generated an average of 15 million reads. Data quality check was done on Illumina SAV. Demultiplexing was performed with Illumina Bcl2fastq2 v 2.17 program.

### Computational analysis

#### Cytokine normalization

Prior to analysis, the cytokine data were separated into two matrices including the serum and plasma samples each. These cytokine measurements included all the available data from both cohorts. For both sets of cytokine measurements, values above the upper limit of detection or below the lower limit of detection were set to be equal to those limits. IL-12p70 had an unusually low limit within cohort 1, and so values were set to the lowest measured value to minimize biased results during normalization. As each cytokine may span a different range of values, measurements were first log transformed and then mean-centered for each cytokine across all patients. Finally, because tensor factorization attempts to explain variation among the data, we divided each matrix by its standard deviation to ensure equal overall variance prior to additional scaling.

#### RNA processing

To reduce cohort-to-cohort differences, raw gene expression measurements were batch corrected via ComBat-seq (46). ComBat-seq adjusted gene expression counts were then converted to transcripts per million (TPM). Measured genes with an average TPM below 1 were removed. Prior to analysis, the TPM values for each gene were mean centered and variance scaled.

#### Enrichment analysis

Enrichment analysis was performed with Enrichr to interpret the transcriptomic significance of each component. For each component, both the 500 most positively and 500 most negatively associated genes were evaluated. Genes were compared against the *ARCHS4_Tissues, Azimuth_Cell_Types_2021, GO_Biological_Process_2023, GO_Cellular_Component_2023, Jensen_TISSUES, KEGG_2021_Human, PanglaoDB_Augmented_2021,* and *Reactome_2022* libraries. Both the (i) *P*-value via Fisher's exact test and (ii) combined score via Enrichr's rank-correction test were derived for each gene set with overlap to a component's associated genes. *P*-values were corrected for multiple hypotheses via the Benjamini–Hochberg procedure. Gene sets with an adjusted *P*-value <0.05 were considered significantly enriched.

#### CIBERSORTx analysis

CIBERSORTx analysis was performed to correlate components with relative immune cell quantities (20). Single cell transcriptomic measurements collected from circulating peripheral blood mononuclear cells were used to create a signature matrix (47). Following ComBat-seq processing and TPM conversion, gene expression measurements were provided to CIBERSORTx. As per CIBERSORTx recommendations, we enabled batch correction and disabled quantile normalization. To evaluate cell-type deconvolution performance, 1000 CIBERSORTx permutations were performed to derive *P*-values corresponding to the probability that inferred cell type fractions would be due to random chance alone; *P*-values were corrected for multiple hypothesis testing via Benjamini–Hochberg. Immune cell quantities were inferred for each patient with RNA-seq measurements. To correlate these quantities with the CMTF components, we evaluated the Pearson correlation between CMTF patient factors and inferred cell quantities. Pearson correlations with *P*-values below 0.05 were considered significantly correlated.

#### DESeq2 analysis

DESeq2 analysis was performed to identify differentially expressed genes prior to CMTF factorization (21). Following DESeq2 recommendations, RNA-seq measurements were batch corrected via ComBat-seq but not transformed to transcripts-per-million prior to differential analysis (46). Log-fold change and *P*-values corresponding to significance of differential in gene expression were recorded; *P*-values were corrected for multiple hypothesis testing via Benjamini–Hochberg. Genes with corrected *P*-values below 0.05 were considered significantly differentially expressed.

#### Coupled matrix-tensor factorization

We separated the data into a three-mode tensor organized by patient, cytokine, and measurement source (serum or plasma) coupled with a matrix of RNA-seq measurements for each patient (Fig. 1B). Tensor operations were performed using Tensorly (48). Here, we reduced the molecular measurements into the sum of *R* Kruskal-formatted components:


$$X_{\mathrm{cytokine}}\approx \overset{R}{\underset{r=1}{\sum }}\;a_{r}\circ b_{r}\circ c_{r}=\;{\hat{\;X}}_{\mathrm{cytokine}}$$

$$X_{\mathrm{RNA}}\approx \overset{R}{\underset{r=1}{\sum }}\;a_{r}\circ d_{r}={\hat{X}}_{\mathrm{RNA}}$$

where $a_{r}$, $b_{r}$, $c_{r}$, and $d_{r}$ are vectors indicating variation along the patient, cytokine species, cytokine sources (serum or plasma), and RNA-seq modes, respectively, and “∘” indicates vector outer product. Concatenating all *R* vectors for each mode, we have their factor respective matrices, *A*, *B*, *C*, and *D*.

As we have reported elsewhere (13), tensor factorization was performed via an alternating censored least squares method (9). Each factor matrix was first initialized with imputed singular value decomposition of the unfolded tensor along its respective mode. Randomized SVD solving was used for sufficient performance given the large size of the dataset when unfolded and including RNA-seq measurements. With each iteration, least squares solving is performed separately for each mode with the missing values ignored. The cytokine factor matrix (*B*) is updated to the least squares solution of the Khatri-Rao product (indicated by “⊙”) of the cytokine source (*C*) and patient (*A*) factors and the cytokine tensor unfolded along the cytokine mode ($X_{\mathrm{cytokine},(2)}$)


$$\underset{B}{\min}\;\|X_{\mathrm{cytokine},(2)}\;-B(C⊙A{)}^{T}{\|}^{2},$$

and the cytokines source matrix is updated in a similar fashion, with the unfolding performed along the source mode ($X_{\mathrm{cytokine},(3)}$)


$$\underset{C}{\min}\;\|X_{\mathrm{cytokine},(3)}\;-C(B⊙A{)}^{T}{\|}^{2}.$$

For the RNA-seq matrix, the RNA factor matrix (*D*) is updated to the least squares solution of the patient factors and RNA-seq matrix


$$\underset{D}{\min}\;\|X_{\mathrm{RNA}}\;-AD^{T}{\|}^{2}.$$

Finally, to enforce that the patient factors explain the variance across both datasets, the unfolded cytokine tensor is concatenated with the RNA-seq matrix


$$\underset{A}{\min}\;\|[X_{\mathrm{cytokine},(1)}\;\;X_{\mathrm{RNA}}]-A[{\left(C⊙B\right)}^{T}\;D^{T}]\;{\|}^{2},$$

where $X_{\mathrm{cytokine},(1)}$ indicates the tensor unfolding of $X_{\mathrm{cytokine}}$ along its patient mode, and “[]” indicates the concatenation of two matrices within the bracket. Similarly, the Khatri-Rao product of the cytokine (*B*) and cytokine source (*C*) factors is concatenated with the RNA-seq factors (*D*) and the least squares solution is derived using these concatenated matrices, leading to patient factors that minimize squared error overall. Iterations were repeated until the variance explained (R2X) improved by less than 1 × 10^−6^ between iterations.

#### Reconstruction fidelity

To evaluate the fidelity of our factorization, we calculate the percent variance explained, R2X. First, the total variance is derived as the sum of the Frobenius norms squared of the cytokine tensor and RNA-seq matrix: $V_{\mathrm{total}}=\;\|X_{\mathrm{cytokine}}{\|}^{2}+X_{\mathrm{RNA}}{\|}^{2}$. We then calculate the Frobenius norms squared of the difference between the cytokine tensor and RNA matrix and their reconstructed versions: $V_{r,\;\mathrm{cytokine}}=\|X_{\mathrm{cytokine}}-{\hat{\;X}}_{\mathrm{cytokine}}{\|}^{2}$ and $V_{r,\mathrm{RNA}}=\|X_{\mathrm{RNA}}-{\hat{X}}_{\mathrm{RNA}}{\|}^{2}$. The variance explained is then calculated as


$$R2X=1-\frac{V_{r,\mathrm{cytokine}}+V_{r,\mathrm{RNA}}}{V_{\mathrm{total}}}$$

Missing values are ignored in all calculations.

#### Balanced accuracy

To address class imbalances in our dataset, we used the balanced accuracy instead of accuracy to evaluate model performances against categorical variables (49). To derive balanced accuracy, we first calculate the recall, or sensitivity, for each discrete class; balanced accuracy is the arithmetic mean of recall scores across classes.

#### Prediction models

We used scikit-learn's logistic regression classifier to predict categorical clinical variables from molecular measurements (50). Data are regularized via elastic-net regularization with an 0.8 L1 ratio. Regularization strength was fitted via grid search using a stratified 10-fold cross-validation to evaluate prediction accuracy. Age, a continuous variable, was predicted using scikit-learn's linear regression model.

Support vector classification models used scikit-learn's implementation with a radial basis function (RBF) kernel. Data were regularized using L2 regularization; regularization strength and RBF complexity (gamma) were fit to optimize balanced accuracy on stratified 10-fold cross-validation.

Principal component analysis (PCA) models used statsmodels's implementation (51); PCA models considered all measurements (serum and plasma cytokines, RNA-seq) concatenated into a single wide matrix. Prior to concatenation, cytokine and RNA-seq measurements were scaled to match variance scaling in the CMTF model. Missing values were imputed via statsmodels's PCA “fill-em” option. Each measurement was set to have zero-mean but not variance scaled to preserve variance scaling between cytokine and RNA-seq measurements. Unless otherwise noted, PCA models used eight components.

## Supplementary Material

pgae185_Supplementary_Data

## Data Availability

The code used to perform these analyses is available on GitHub at https://github.com/meyer-lab/tfac-mrsa. This repository also includes all the cytokine and RNA-seq count measurements, as well as the enrichment results for genes most positively and negatively associated with each component. RNA-seq read measurements are deposited within the Sequence Read Archive, NCBI project PRJNA914756.
