# Supplementary material for: Tensor modeling of MRSA bacteremia cytokine and transcriptional patterns reveals coordinated, outcome-associated immunological programs
Source: PNAS Nexus. 2024 May 4;3(5):pgae185. doi: 10.1093/pnasnexus/pgae185 (PMC11109816; doi:10.1093/pnasnexus/pgae185)
Supplement: pgae185_Supplementary_Data [file pgae185_supplementary_data.zip › PNASNEXUS-PNASNEXUS-2023-01274-TR-s01.docx]

**Supporting Information for**

Tensor modeling of MRSA bacteremia cytokine and transcriptional patterns reveals coordinated, outcome-associated immunological programs

Jackson L. Chin, Zhixin Cyrillus Tan, Liana C. Chan, Felicia Ruffin, Rajesh Parmar, Richard Ahn, Scott Taylor, Arnold S. Bayer, Alexander Hoffmann, Vance G. Fowler, Jr.^†^, Elaine F. Reed^†^, Michael R. Yeaman^†^, Aaron S. Meyer*, with the MRSA Systems Immunobiology Group

^†^Equivalent senior authors.

*Corresponding author: Aaron S. Meyer

Email: ameyer@asmlab.org

**This PDF file includes:**

Figures S1 to S5

**Figure S1. Cytokine measurements vary by source and persistence status.** A) Pearson’s correlation coefficient between plasma and serum cytokine measurements for each measured cytokine. B–C) Boxplots depicting normalized serum (B) and plasma (C) cytokine measurements. Boxplots for each cytokine are separated by persistence outcome.

**Figure S2. Correlations between cytokines.** Pearson’s correlation coefficients between cytokine species for serum (A) and plasma (B) cytokine measurements.

**Figure S3. CMTF components more effectively assign persistence outcome than PCA components.** A) Balanced accuracy in RB/PB assignment for models trained with CMTF components compared against models trained with PCA components and with raw data sources with missing values imputed via PCA. Accuracy is evaluated using 10-fold cross-validation over the training cohort. B) Receiver operating characteristic curves for the models depicted in (A). C) Balanced accuracy in validation cohort RB/PB assignment for models in (A). Model accuracy is evaluated against a masked validation cohort following training against the training cohort. D) Receiver operating characteristic curves for models depicted in (C).

**Figure S4. Data missingness.** Heatmaps depicting available data-types for each patient, subset by cohort.

**Figure S5. CMTF transcriptomic factors are unique from differentially-expressed genes.** A-C) Transcriptomic factors for components 2, 4, and 6 compared against differential expression p-values. The 500 genes most positively- and most negatively-associated with each component are colored in red (component 2), green (component 4), and cyan (component 6). The dashed line represents a p-value of 0.05; genes above this line are significantly differentially-expressed via DESeq2.
