# Supplementary figures and images for: Tensor modeling of MRSA bacteremia cytokine and transcriptional patterns reveals coordinated, outcome-associated immunological programs
Source: PNAS Nexus. 2024 May 4;3(5):pgae185. doi: 10.1093/pnasnexus/pgae185 (PMC11109816; doi:10.1093/pnasnexus/pgae185)

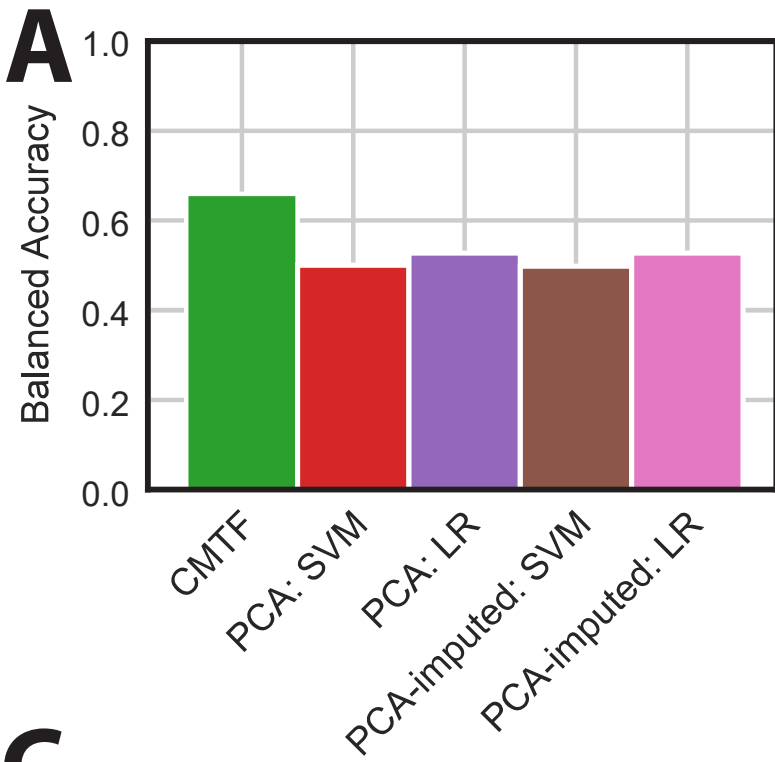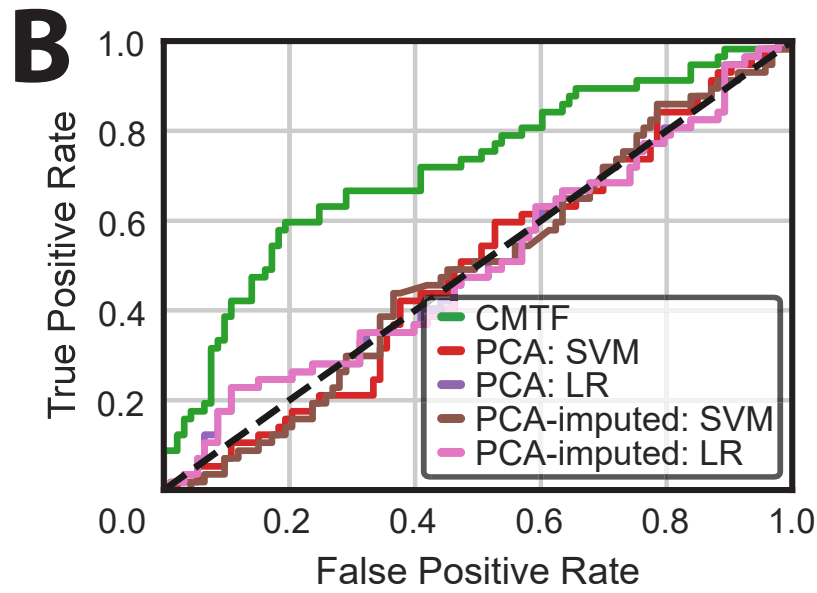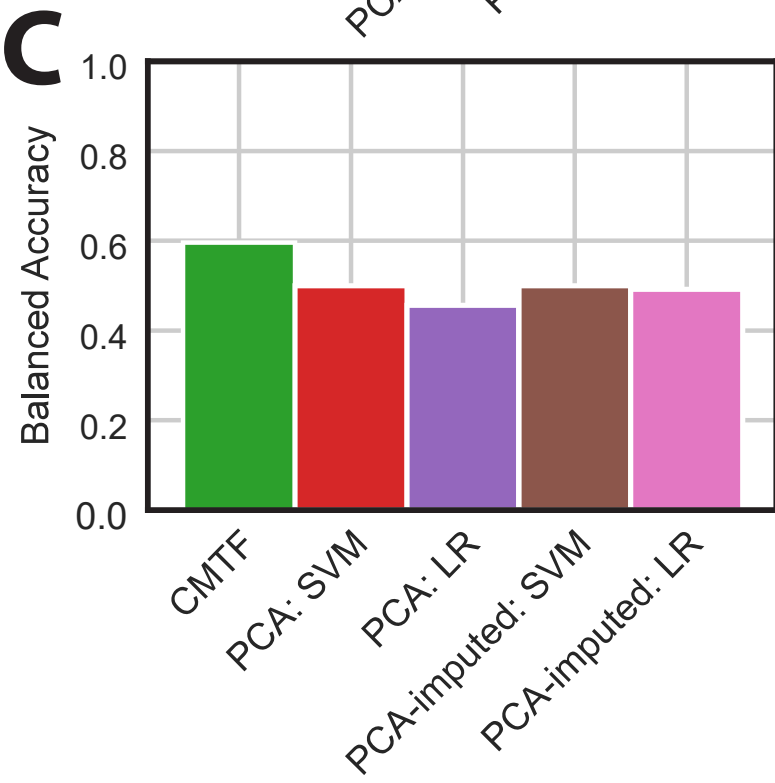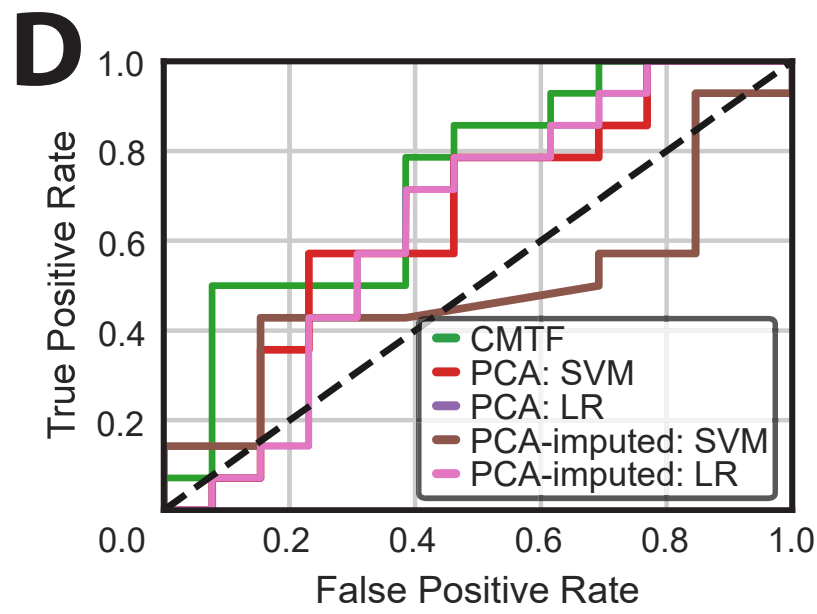

Supplement: pgae185_Supplementary_Data [file pgae185_supplementary_data.zip › PNASNEXUS-PNASNEXUS-2023-01274-TR-s04.pdf]

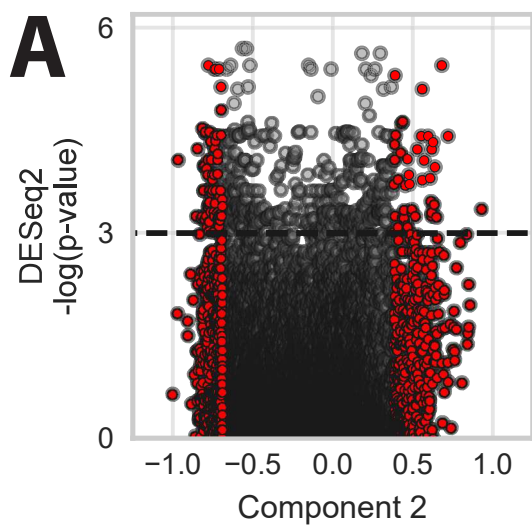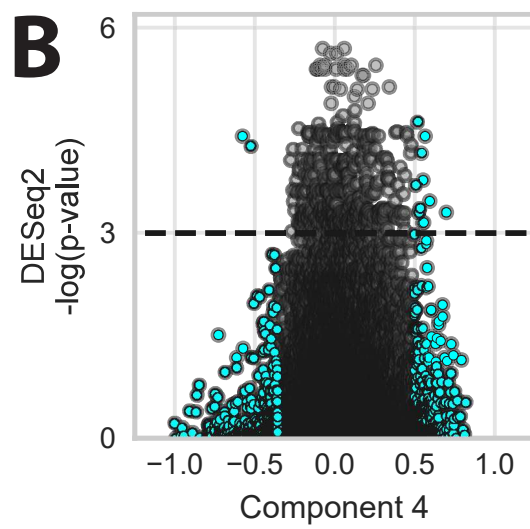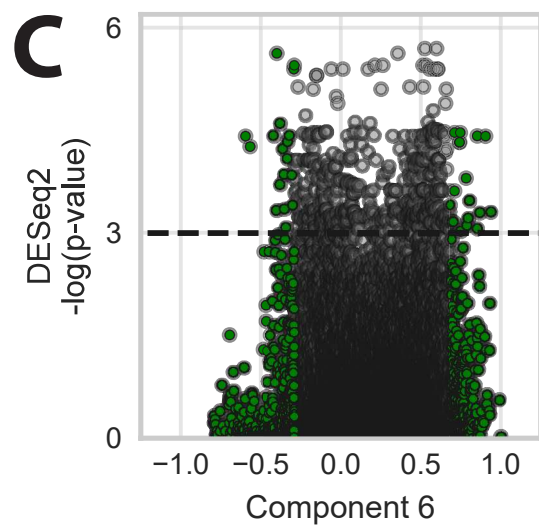

Supplement: pgae185_Supplementary_Data [file pgae185_supplementary_data.zip › PNASNEXUS-PNASNEXUS-2023-01274-TR-s06.pdf]

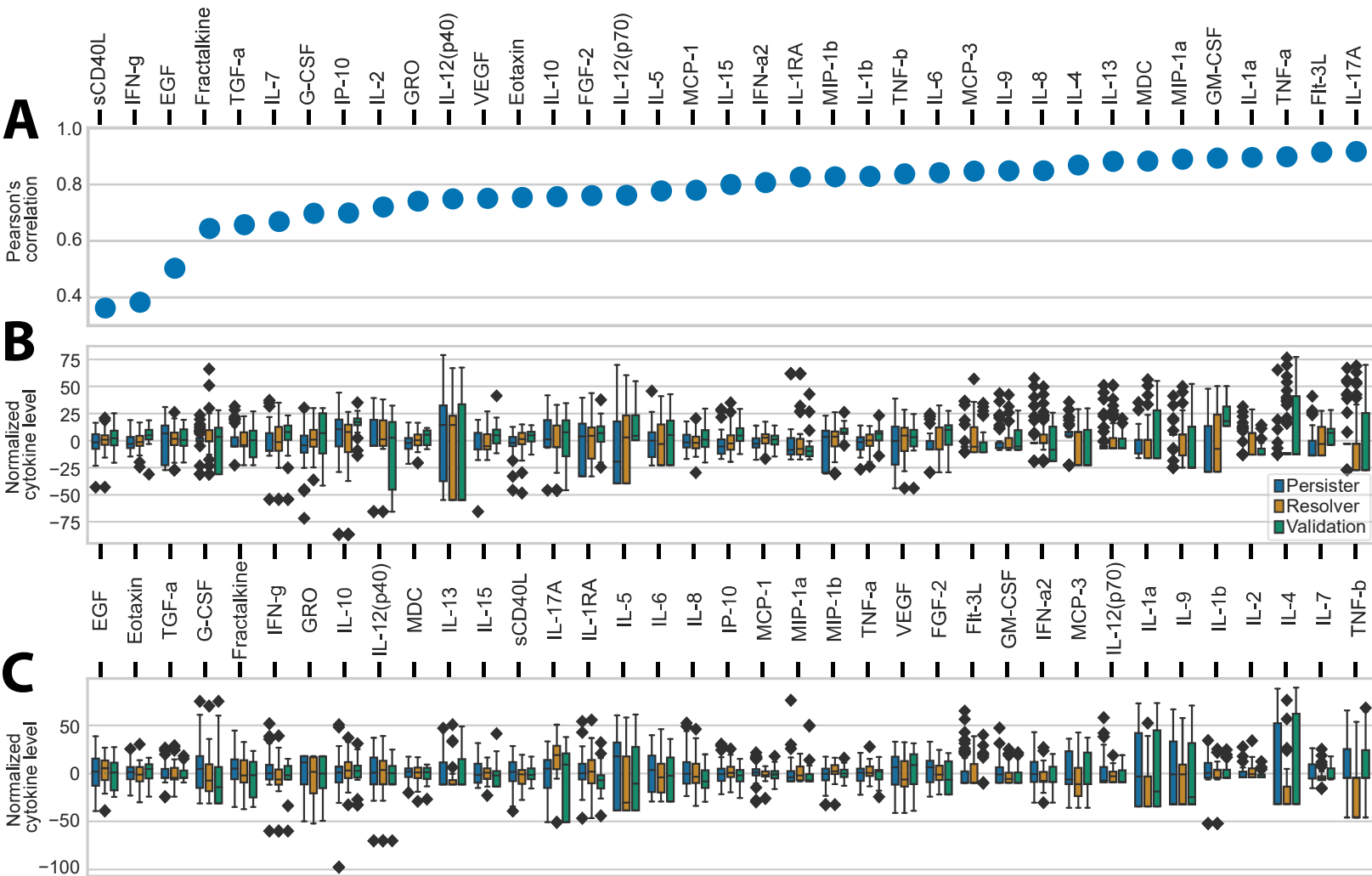

Supplement: pgae185_Supplementary_Data [file pgae185_supplementary_data.zip › PNASNEXUS-PNASNEXUS-2023-01274-TR-s02.pdf]

**A**

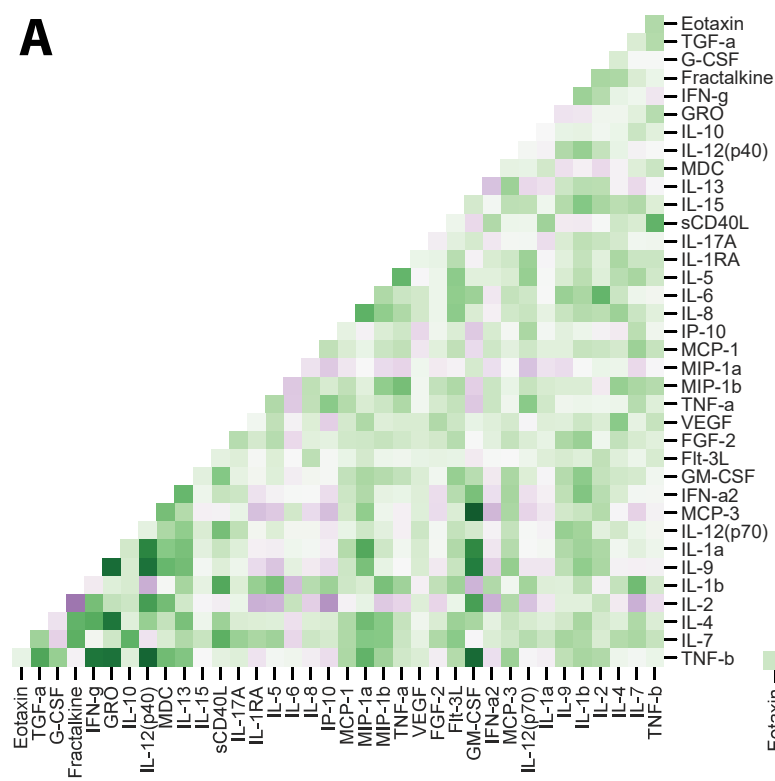

**B**

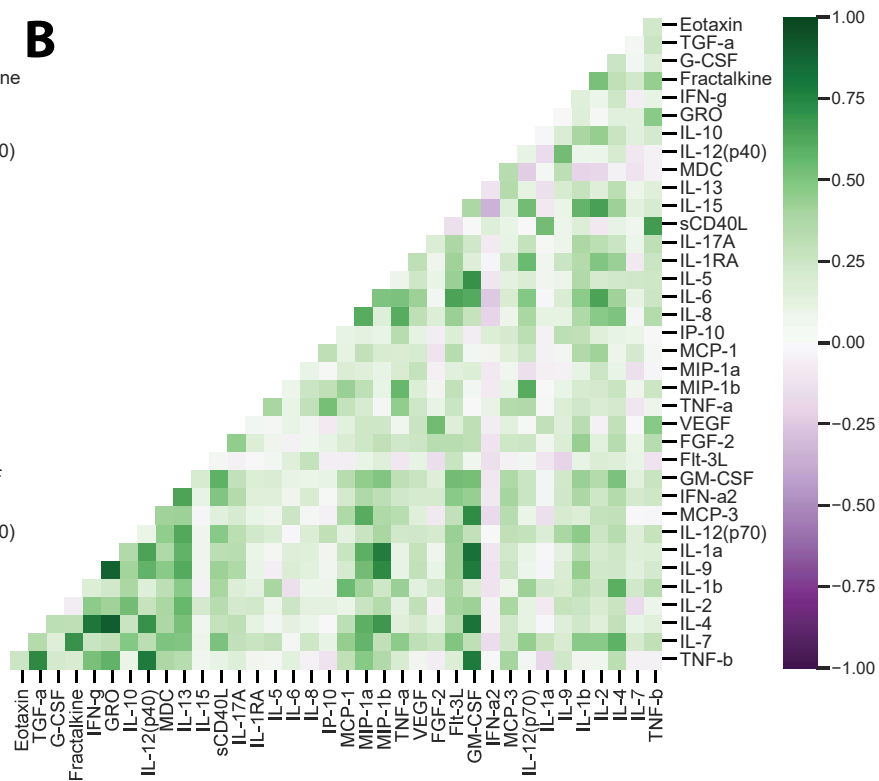

Supplement: pgae185_Supplementary_Data [file pgae185_supplementary_data.zip › PNASNEXUS-PNASNEXUS-2023-01274-TR-s03.pdf]
