# Supplementary material for: Tensor modeling of MRSA bacteremia cytokine and transcriptional patterns reveals coordinated, outcome-associated immunological programs
Source: PNAS Nexus. 2024 May 4;3(5):pgae185. doi: 10.1093/pnasnexus/pgae185 (PMC11109816; doi:10.1093/pnasnexus/pgae185)
Supplement: pgae185_Supplementary_Data [file PNASNEXUS-PNASNEXUS-2023-01274-TR-s05.pdf › PNASNEXUS-PNASNEXUS-2023-01274-TR-s05.pdf]

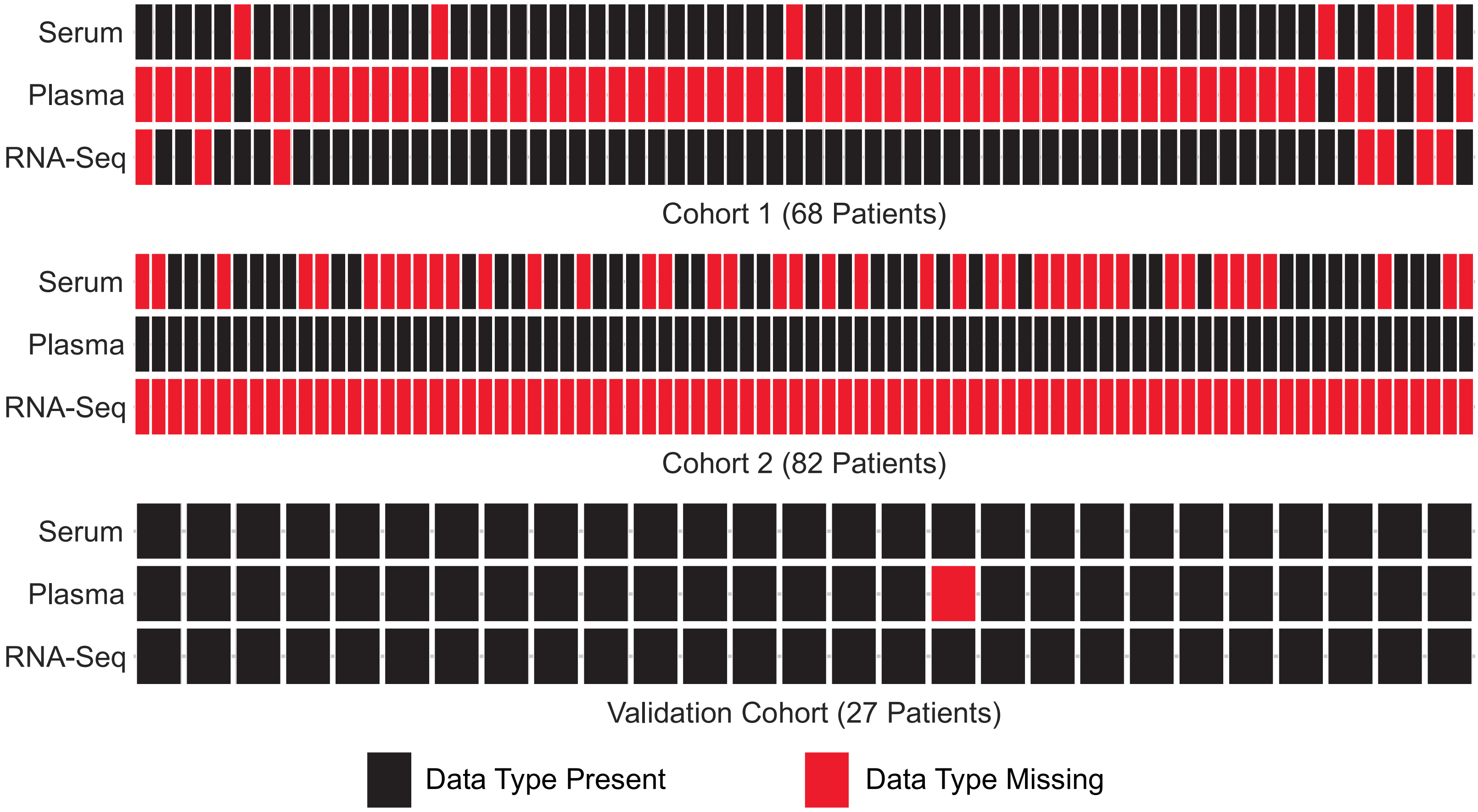
Serum
Plasma
RNA-Seq
Cohort 1 (68 Patients)
Serum
Plasma
RNA-Seq
Cohort 2 (82 Patients)
Serum
Plasma
RNA-Seq
Validation Cohort (27 Patients)
Data Type Present
Data Type Missing
